# GRaSP-web: a machine learning strategy to predict binding sites based on residue neighborhood graphs

**DOI:** 10.1093/nar/gkac323

**Published:** 2022-05-07

**Authors:** Charles A Santana, Sandro C Izidoro, Raquel C de Melo-Minardi, Jonathan D Tyzack, António J M Ribeiro, Douglas E V Pires, Janet M Thornton, Sabrina de A. Silveira

**Affiliations:** Department of Biochemistry and Immunology, Universidade Federal de Minas Gerais, Belo Horizonte 31270-901, Brazil; Department of Computer Science, Universidade Federal de Minas Gerais, Belo Horizonte 31270-901, Brazil; Institute of Technological Sciences (ICT), Advanced Campus at Itabira, Universidade Federal de Itajubá, Itabira 35903-087, Brazil; Department of Biochemistry and Immunology, Universidade Federal de Minas Gerais, Belo Horizonte 31270-901, Brazil; Department of Computer Science, Universidade Federal de Minas Gerais, Belo Horizonte 31270-901, Brazil; European Molecular Biology Laboratory, European Bioinformatics Institute, Wellcome Trust Genome Campus, Hinxton, Cambridge CB10 1SD, UK; European Molecular Biology Laboratory, European Bioinformatics Institute, Wellcome Trust Genome Campus, Hinxton, Cambridge CB10 1SD, UK; School of Computing and Information Systems, University of Melbourne, Parkville 3052, Australia; European Molecular Biology Laboratory, European Bioinformatics Institute, Wellcome Trust Genome Campus, Hinxton, Cambridge CB10 1SD, UK; Department of Computer Science, Universidade Federal de Viçosa, Viçosa 36570-900, Brazil

## Abstract

Proteins are essential macromolecules for the maintenance of living systems. Many of them perform their function by interacting with other molecules in regions called binding sites. The identification and characterization of these regions are of fundamental importance to determine protein function, being a fundamental step in processes such as drug design and discovery. However, identifying such binding regions is not trivial due to the drawbacks of experimental methods, which are costly and time-consuming. Here we propose GRaSP-web, a web server that uses GRaSP (Graph-based Residue neighborhood Strategy to Predict binding sites), a residue-centric method based on graphs that uses machine learning to predict putative ligand binding site residues. The method outperformed 6 state-of-the-art residue-centric methods (MCC of 0.61). Also, GRaSP-web is scalable as it takes 10-20 seconds to predict binding sites for a protein complex (the state-of-the-art residue-centric method takes 2-5h on the average). It proved to be consistent in predicting binding sites for bound/unbound structures (MCC 0.61 for both) and for a large dataset of multi-chain proteins (4500 entries, MCC 0.61). GRaSPWeb is freely available at https://grasp.ufv.br.

## Graphical Abstract

**Graphical Abstract:**
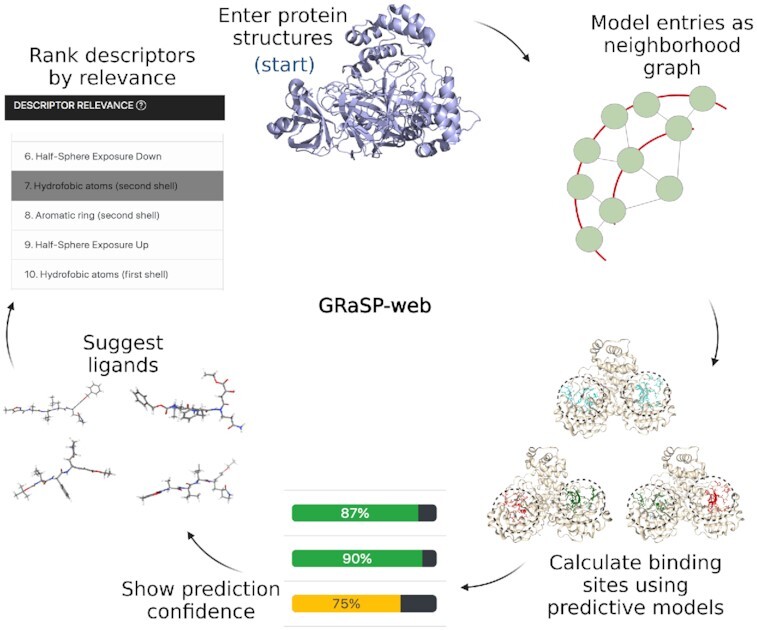
GRaSP-web: a machine learning strategy to predict binding sites based on residue neighborhood graphs.

## INTRODUCTION

Proteins perform their roles through interactions with other molecules, including organic compounds, nucleotides, metal ions, and even other proteins. The biological function of a large number of proteins is still unknown (1), with the knowledge of the binding sites, as well as the identification of amino acid residues involved in ligand binding, being a crucial step for protein functional characterization (2). However, determining protein binding sites through *in vitro* and *in vivo* experimental methods is expensive and time-consuming.

To address this issue, a range of structure based methods have been developed to identify protein binding site residues. Here we present some representative examples. Firestar (3,4) identifies ligand binding residues based on local sequence conservation matches to known ligand-binding residues in FireDB (5). FunFold3 (6,7) points binding residues performing a superimposition of structural templates containing relevant ligands on the target model. COACH (8,9) is a consensus method that combines their in-house developed algorithms TM-SITE and S-SITE, with third-party prediction tools COFACTOR (10), FINDSITE (11) and ConCavity (12) using a supervised learning algorithm. In LigDig (13), ligands are the starting point for binding site prediction, combining databases in a ligand interaction network to identify similar ligands and their binding proteins, which allows to point potential protein–ligand binding sites. GASS (14,15) proposes a genetic algorithm that searches for active site structural templates from CSA (16) in unknown proteins.

Many of the recent developments in the field suffer from limitations such as prohibitive execution time, lack of support for binding sites at protein interfaces and absence of explainable and visual results. Here, we present GRaSP-web, a scalable and user-friendly web server for the prediction of protein-ligand binding sites. GRaSP-web provides a web interface to GRaSP (Graph-based Residue neighborhood Strategy to Predict binding sites) (17), which is a computational strategy that represents a particular residue and its neighbors as a graph at atomic level to perceive residue environment, and use supervised learning to predict residue ligand binding sites.

## MATERIALS AND METHODS

### GRaSP strategy

In GRaSP, the problem of predicting residues that are part of a ligand-binding site is modeled as a binary classification, which aims to predict, for each residue, if it is in the binding site or not. The supervised learning strategy is trained using a data matrix, G, in which each row represents a residue, *r*, and each column encodes a descriptor. Thus each residue, *r*, is encoded as a feature vector, and a whole protein structure, *P*, is encoded as a set of feature vectors.

The feature vectors that encode each residue are composed of descriptors calculated at: (i) residue level, which are solvent relative accessibility and cysteine; (ii) atom level, in which we compute how many atoms of each type each residue presents. The atoms are labeled as aromatic, acceptor, donor, hydrophobic, positive, negative; (iii) interaction level, in which non-covalent interactions are calculated based on the type of atoms and on the Euclidean distance between them. The interaction types calculated are aromatic stacking, disulfide bridge, hydrogen bond, hydrophobic, repulsive and salt bridge. This totals 14 descriptors.

This set properties (the 14 descriptors) are calculated for the residue being considered, for its first and for its second shell of neighbor residues. To summarize each neighbor shell, descriptors are averaged by the number of residues in the shell. This builds our graph model that captures physicochemical properties of the structural environment of each residue. Hence, each feature vector is composed of 42 descriptors, which means that there are 42 descriptors to represent each residue. GRaSP is a residue-centric strategy to predict binding sites that models each residue and its first two shells of neighbors as a graph in order to perceive physicochemical environment information. For each residue of a protein, GRaSP encodes it into numerical descriptors, in which topological and physicochemical properties of its atoms and interactions are represented as a graph which, in turn, are encoded as a feature vector. The set of feature vectors is used to train predictive models. For more details on problem modeling, please refer to (17).

To predict residue ligand binding sites GRaSP performs a supervised learning task using the Extremely Randomized Trees algorithm, that belongs to a class of ensemble classifiers, implemented in scikit-learn (scikit-learn.org). GRaSP was compared with methods described in the literature, achieving comparable or higher prediction metrics against six other residue-centric methods. Additionally, our method took 10–20 s on average to predict the binding site for a protein complex, whereas the state-of-the-art residue-centric method takes 2–5 h on average. Another advantage of GRaSP is the ability to predict protein binding sites from multi-chain structures;, hence it is able to find binding sites even at protein-protein interfaces.

### Webserver

**Figure 1.**
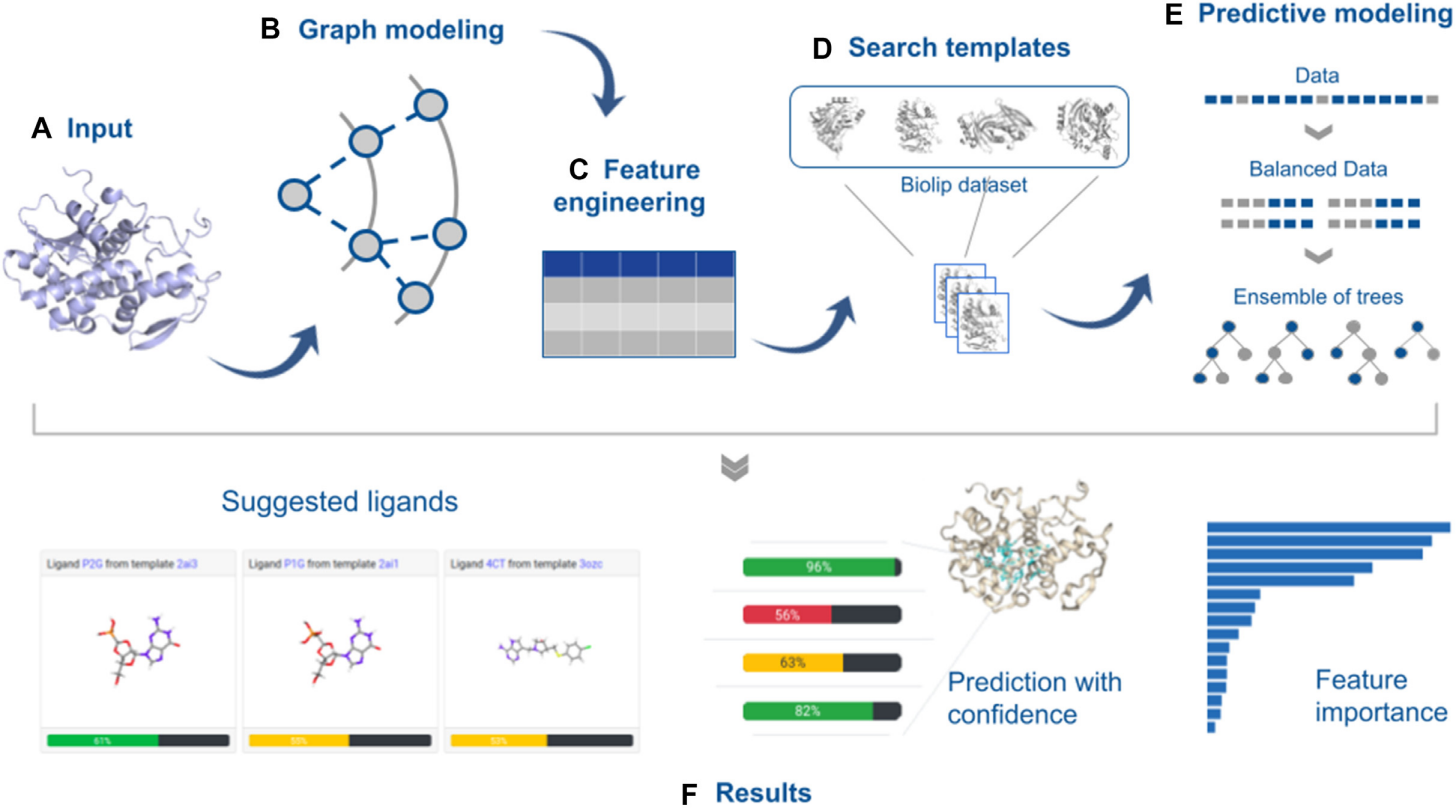
GRaSP-web workflow. In (**A**) we see an input protein. It is modeled as a neighborhood graph in (**B**), which is encoded as a feature vector (**C**). A training dataset is built with data extracted from the BioLip database in (**D**). Binding site residues are predicted by an ensemble classifier in (**E**). In (**F**), the results are presented: potential binding sites, suggested ligands and ranked feature importance. This figure was inspired by aCSM workflow (19).

GRaSP-web provides a web interface enabling users to easily access the predictions visually. The server intuitive front-end was built using the Bootstrap framework, while the back-end was built using the Flask framework for Python running on Apache server, and the communication is made using a web server gateway interface described in Python Enhancement Proposal 333. The GRaSP-web processing steps are shown in Figure 1. Each residue of an input protein is modeled as a neighborhood graph , which is encoded as a feature vector. Then, a training dataset is built from proteins in the BioLip database (18) that are similar to the input protein. Next, an ensemble of balanced classifiers is used to predict the residues of the input protein that are part of a binding site. As results, the web server presents the predicted binding site residues in a molecular viewer, coupled with confidence scores, that represent how confident the method is for each prediction; residues can also be clustered in binding sites and potential ligands are suggested for each of these sites; the ranked relative importance of descriptors is also presented which can be inspected to support users on the understanding of predictions.

### Input

To perform the prediction, users can upload one or many three-dimensional structures in the PDB format or provide one or more PDB identification codes, in which case GRaSP-web will retrieve and store the corresponding structures from the PDB database (20).

The query structure is submitted to predict the ligand binding sites (Figure 1A). When the protein is submitted, GRaSP uses graph modeling to perceive the chemical environment of each residue and encode it into numerical features (Figure 1B, C). These feature vectors are combined into a matrix which is used for the ligand-binding site prediction (Figure 1C). The amino acid sequence of the query structure is matched with the ligand-binding templates from BioLip (18). The BioLip is a semi-manually curated database for biologically relevant ligand-protein interactions constructed based on the PDB database. These templates are used as training data by our method to build the supervised machine learning model.

Data imbalance is intrinsic to the problem of predicting binding sites. The number of non-binding site residues is greater than the number of binding site residues, which decreases the predictive power of conventional classifiers. So GRaSP-web combines a resampling approach with an ensemble of trees to balance the data and improve the predictions (Figure 1E). GRaSP-web takes about 20 seconds to process a protein complex with ∼300 residues. Moreover, we aim to make predictions more informative by taking advantage of the white box model in decision trees (Figure 1F). For example, the prediction confidence, which means the class probabilities of the trees, can be used to verify how homogeneous the ensemble prediction was. Furthermore, we can rank the set of features according to its relative importance with respect to the predictability of the target variable.

### Output

**Figure 2.**
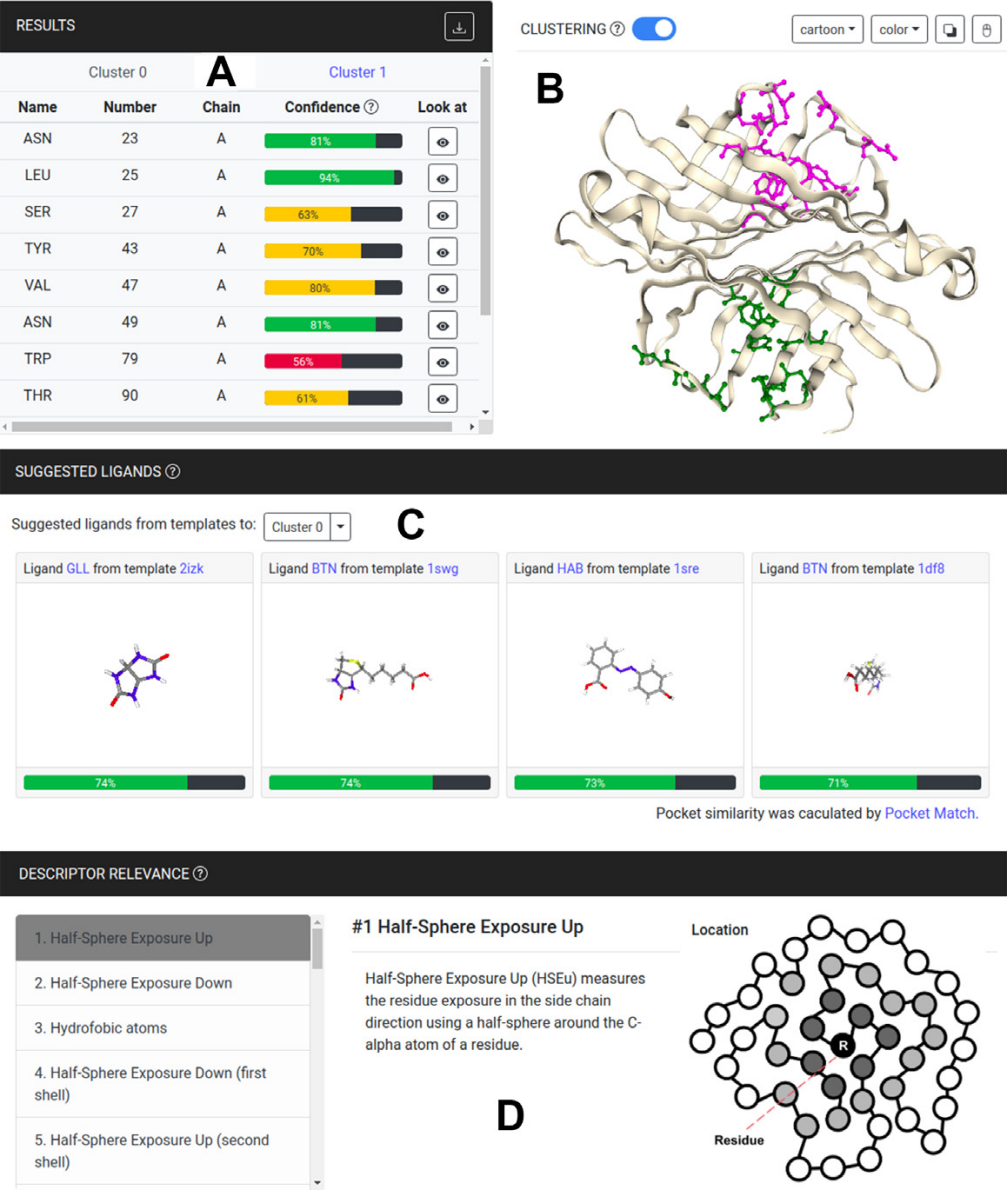
GRaSP-web results page. Binding site residues are presented coupled with confidence scores in (**A**). A molecular viewer shows in (**B**) binding site residues clustered as potential binding sites. A set of ligands are suggested for each binding site in (**C**). The relative importance of descriptors is presented in (**D**).

A URL is assigned to each submission so the user can access the results or track the processing status. The standard output for each protein submitted includes a list of predicted ligand binding site residues (Figure 2A), which can be downloaded to a local computer. Each predicted residue has a confidence score, provided to show the reliability of each prediction and computed as the mean predicted class probabilities of the trees in the forest. Additionally, GRaSP-web uses NGL viewer (nglviewer.org) for molecular visualization, where the query protein is shown and the binding site residues are highlighted (Figure 2B).

The GRaSP algorithm is a residue-centric method that models the ligand-binding site prediction problem as a binary classification, which aims to predict, for each residue, whether it is in the binding site or not. In order to give our method a pocket-centric perspective, we provide a way to cluster the predicted residues into groups, emulating pockets. By turning on the ‘clustering’ button (Figure 2B), the set of residues predicted as binding sites are clustered using DBSCAN (21), a density-based clustering algorithm that separates areas of high density from areas of low density.

By clustering the residues into regions that are potential pockets, we can use these subsets of residues to estimate candidate ligands to bind to a specific region (Figure 2(C)). Using the PocketMatch (22) algorithm to compare each cluster of residues against the entire database of binding sites used as training dataset, we can estimate which ligands have the potential to bind to a cluster, since similar binding sites tend to bind to similar ligands. A score bar is provided for each ligand candidate, that represents the PocketMatch comparison score between the respective cluster and the binding site from the template database.

Taking advantage of decision tree explainability, we can rank the set of features used in the prediction of binding site residues according to its relative importance. It is important to highlight that relative importance does not imply causation. The features are ranked and followed by a brief description as shown in Figure 2(D).

## RESULTS

In order to assess the GRaSP-web performance, we used a variety of datasets that captured different characteristics of protein structures, including drug-like complexes, proteins on bound/unbound state and multimeric structures (Supplementary Table S1). The GRaSP strategy was previously assessed on these data and achieved comparable or superior results in comparison with other residue-centric methods (17). As a way to measure the predictive performance of the assessed methods, the metrics Matthews correlation coefficient (MCC), precision, recall, binding distance test (BDT) and distance between the center of the pocket and any ligand atom (DCA) were used. MCC is a correlation coefficient between observed and predicted classifications and it is considered by authors of the state-of-the-art residue centric method as the main measure to compare their strategy with other methods (8). For more details on the evaluation strategy, please refer to (17).

We used the benchmark dataset from COACH (8), a set of 500 non-redundant single-chain proteins, as well as results reported in (9), to compare our method with the state-of-the-art residue centric method (Supplementary Table S2). GRaSP achieved a MCC of 0.61, requiring between 10 and 20 seconds to perform a prediction, whereas the competitor achieved a MCC of 0.60 taking 2–5 h to predict proteins of similar sizes. On the CASP10 (23) dataset, GRaSP achieved an MCC of 0.58, ranking seventh among 18 methods (according to CASP10, differences among the first 10 methods were not statistically significant). It is important to mention that on the CASP10 dataset, GRaSP used as training data just the 25 templates provided for each protein target, while other methods used curated databases, coupled with sequence and structural alignment. Our method outperformed the method from CAMEO (24) independent assessment that resembles GRaSP, showing better average values (MCC 0.656 and BDT 0.632 ) than method Raptor-X-Binding (MCC 0.557 and BDT 0.546). GRaSP ranked second when compared with five state-of-the-art pocket centric methods (DeepSite (25), Fpocket (26), Metapocket 2.0 (27), P2Rank (28,29), Sitehound (30)), which we consider a significant result as our method was not devised to predict pockets that are potential binding sites.

**Figure 3.**
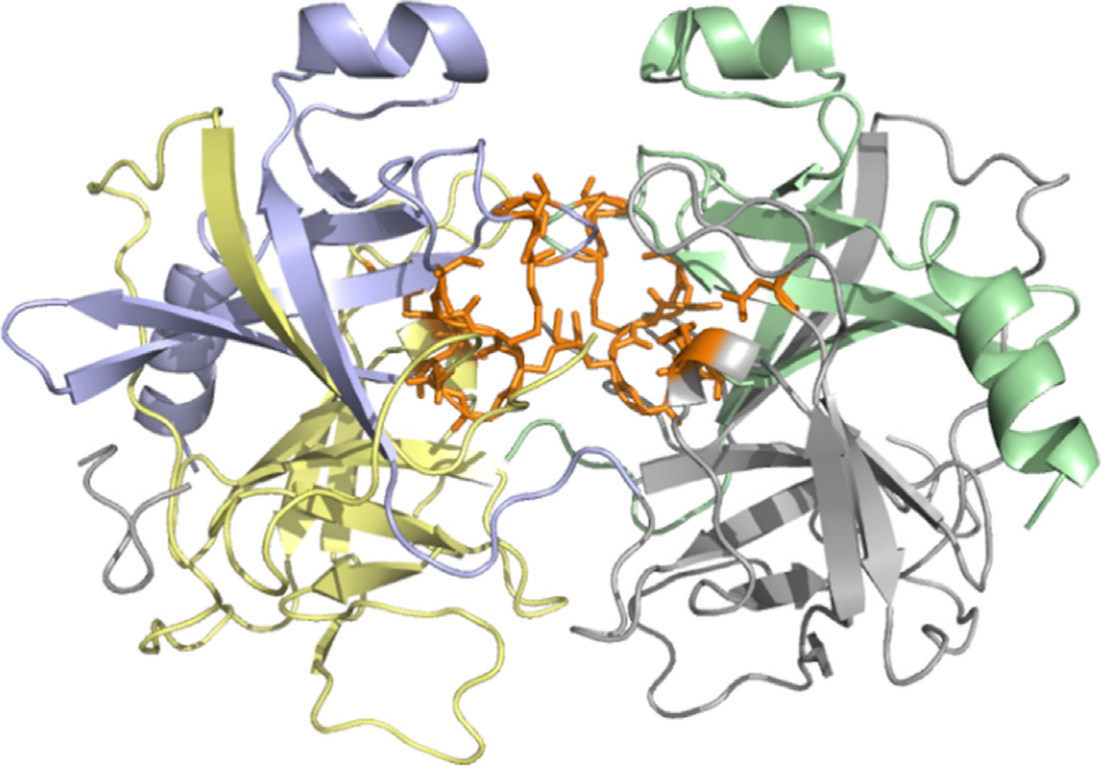
Binding site residues predicted by GRaSP-web (in orange) for the multiple chain protein structure of alpha-chymotrypsin (PDB: 2CHA).

Since the method considered state-of-the-art is not able to process multi-chain structures, we perform additional experiments using a diverse dataset containing single chain and multiple chains protein structures. As a result, GRaSP-web proved to be consistent in predicting ligand-binding site residues at protein-protein interfaces. Figure 3 shows the structure of alpha-chymotrypsin (PDB: 2CHA), a hetero hexamer, with its binding site between chains predicted by GRaSP-web, evidencing that our method is able to predict residue binding sites in this context.

**Figure 4.**
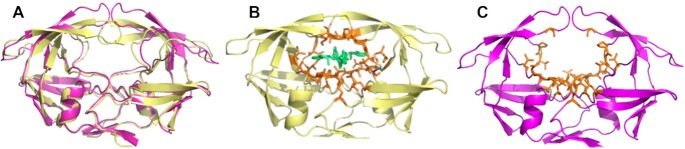
Protein structure of HIV protease. (**A**) Superposition of the HIV protease structures 4PHV (bound state in beige) and 3PHV (unbound state in magenta). (**B**, **C**) Prediction performed by GRaSP-web in orange in both conformational states of HIV protease.

Another aspect that needs to be highlighted when predicting binding site residues is the conformational state of the protein. Many proteins undergo induced fit when binding to ligand molecules, changing the conformation of the protein structure. Binding site prediction methods should ignore these variations in protein folding, focusing only on physicochemical patterns of the cavity in both bound and unbound states. We used a benchmark containing 44 protein structures in both states, obtained from (28), totaling 88 entries, to assess whether GRaSP-web is able to predict residue binding sites correctly (Supplementary Table S3). Figure 4 shows the structure of the HIV protease in complex with the VAC ligand (pdb 4PHV), and the HIV protease in unbound state (pdb 3PHV). GRaSP-web was able to correctly predict the binding site residues in both scenarios, achieving an overall average MCC of 0.67 for the bound/unbound benchmark.

## CONCLUSION

In this work, we introduced the GRaSP-web server, a user-friendly and visual interface to GRaSP, which is a residue-centric approach to predict ligand-binding sites. Each amino acid residue, as well as its neighborhood of residues, is modeled as a graph that captures the physicochemical properties of the residue environment. All information is encoded as a feature vector that serves as input to a supervised machine learning strategy. Experiments performed with several benchmarks showed that our server is capable of predicting binding site residues with compatible or superior results to existing methods. In addition to its speed, GRaSP-web was able to correctly predict binding sites for heterogeneous structural data, contemplating protein structures which are druggable, containing single and multiple chains, and featuring distinct conformational states, such as bound and unbound structures. The users can submit their own protein structures, or structures from the PDB database. After that, binding site residues are predicted, and GRaSP-web provides a user-friendly interface along with visual insights into the predictions made. We believe that these features incorporated by GRaSP-web can contribute to protein function prediction studies and to the discovery or development of new drugs.

## DATA AVAILABILITY

GRaSP-web strategy is available as a web server at https://grasp.ufv.br/. The source code and datasets are available at https://github.com/charles-abreu/GRaSP.

## Supplementary Material

gkac323_Supplemental_File

## References

[1] Mistry J. , Chuguransky S., Williams L., Qureshi M., Salazar G.A., Sonnhammer E.L., Tosatto S.C., Paladin L., Raj S., Richardson L.J. et al. Pfam: The protein families database in 2021. Nucleic Acids Res. 2021; 49:D412–D419. 33125078 10.1093/nar/gkaa913 PMC7779014

[2] Zhao J. , Cao Y., Zhang L. Exploring the computational methods for protein-ligand binding site prediction. Comput. Struct. Biotechnol. J. 2020; 18:417–426. 32140203 10.1016/j.csbj.2020.02.008 PMC7049599

[3] López G. , Valencia A., Tress M.L. firestar—prediction of functionally important residues using structural templates and alignment reliability. Nucleic Acids Res. 2007; 35:W573–W577. 17584799 10.1093/nar/gkm297 PMC1933227

[4] Lopez G. , Maietta P., Rodriguez J.M., Valencia A., Tress M.L. firestar—advances in the prediction of functionally important residues. Nucleic Acids Res. 2011; 39:W235–W241. 21672959 10.1093/nar/gkr437 PMC3125799

[5] Lopez G. , Valencia A., Tress M. FireDB—a database of functionally important residues from proteins of known structure. Nucleic Acids Res. 2007; 35:D219–D223. 17132832 10.1093/nar/gkl897 PMC1716728

[6] Roche D.B. , Tetchner S.J., McGuffin L.J. FunFOLD: an improved automated method for the prediction of ligand binding residues using 3D models of proteins. BMC bioinformatics. 2011; 12:160. 21575183 10.1186/1471-2105-12-160 PMC3123233

[7] Roche D.B. , Buenavista M.T., McGuffin L.J. The FunFOLD2 server for the prediction of protein–ligand interactions. Nucleic Acids Res. 2013; 41:W303–W307. 23761453 10.1093/nar/gkt498 PMC3692132

[8] Yang J. , Roy A., Zhang Y. Protein–ligand binding site recognition using complementary binding-specific substructure comparison and sequence profile alignment. Bioinformatics. 2013; 29:2588–2595. 23975762 10.1093/bioinformatics/btt447 PMC3789548

[9] Wu Q. , Peng Z., Zhang Y., Yang J. COACH-D: improved protein–ligand binding sites prediction with refined ligand-binding poses through molecular docking. Nucleic Acids Res. 2018; 46:W438–W442. 29846643 10.1093/nar/gky439 PMC6030866

[10] Roy A. , Yang J., Zhang Y. COFACTOR: an accurate comparative algorithm for structure-based protein function annotation. Nucleic Acids Res. 2012; 40:W471–W477. 22570420 10.1093/nar/gks372 PMC3394312

[11] Brylinski M. , Skolnick J. A threading-based method (FINDSITE) for ligand-binding site prediction and functional annotation. Proc. Nat. Acad. Sci. U.S.A. 2008; 105:129–134. 10.1073/pnas.0707684105 PMC2224172 18165317

[12] Capra J.A. , Laskowski R.A., Thornton J.M., Singh M., Funkhouser T.A. Predicting protein ligand binding sites by combining evolutionary sequence conservation and 3D structure. PLoS Comput. Biol. 2009; 5:e1000585. 19997483 10.1371/journal.pcbi.1000585 PMC2777313

[13] Fuller J.C. , Martinez M., Henrich S., Stank A., Richter S., Wade R.C. LigDig: a web server for querying ligand–protein interactions. Bioinformatics. 2015; 31:1147–1149. 25433696 10.1093/bioinformatics/btu784 PMC4382906

[14] Izidoro S.C. , de Melo-Minardi R.C., Pappa G.L. GASS: identifying enzyme active sites with genetic algorithms. Bioinformatics. 2015; 31:864–870. 25388152 10.1093/bioinformatics/btu746

[15] Moraes J.P. , Pappa G.L., Pires D.E., Izidoro S.C. GASS-WEB: a web server for identifying enzyme active sites based on genetic algorithms. Nucleic Acids Res. 2017; 45:W315–W319. 28459991 10.1093/nar/gkx337 PMC5570142

[16] Porter C.T. , Bartlett G.J., Thornton J.M. The Catalytic Site Atlas: a resource of catalytic sites and residues identified in enzymes using structural data. Nucleic Acids Res. 2004; 32:D129–D133. 14681376 10.1093/nar/gkh028 PMC308762

[17] Santana C.A. , Silveira S. D.A., Moraes J.P., Izidoro S.C., de Melo-Minardi R.C., Ribeiro A.J., Tyzack J.D., Borkakoti N., Thornton J.M. GRaSP: a graph-based residue neighborhood strategy to predict binding sites. Bioinformatics. 2020; 36:i726–i734. 33381849 10.1093/bioinformatics/btaa805

[18] Yang J. , Roy A., Zhang Y. BioLiP: a semi-manually curated database for biologically relevant ligand–protein interactions. Nucleic Acids Res. 2012; 41:D1096–D1103. 23087378 10.1093/nar/gks966 PMC3531193

[19] Pires D.E. , de Melo-Minardi R.C., Da Silveira C.H., Campos F.F., Meira Jr W. aCSM: noise-free graph-based signatures to large-scale receptor-based ligand prediction. Bioinformatics. 2013; 29:855–861. 23396119 10.1093/bioinformatics/btt058

[20] Rose P.W. , Prlić A., Altunkaya A., Bi C., Bradley A.R., Christie C.H., Costanzo L.D., Duarte J.M., Dutta S., Feng Z. et al. The RCSB protein data bank: integrative view of protein, gene and 3D structural information. Nucleic Acids Res. 2016; gkw1000. 10.1093/nar/gkw1000 PMC5210513 27794042

[21] Schubert E. , Sander J., Ester M., Kriegel H.P., Xu X. DBSCAN revisited, revisited: why and how you should (still) use DBSCAN. ACM Trans. Database Syst. (TODS). 2017; 42:1–21.

[22] Yeturu K. , Chandra N. PocketMatch: a new algorithm to compare binding sites in protein structures. BMC Bioinformatics. 2008; 9:543. 19091072 10.1186/1471-2105-9-543 PMC2639437

[23] Gallo Cassarino T. , Bordoli L., Schwede T. Assessment of ligand binding site predictions in CASP10. Proteins: Struct. Funct. Bioinformatics. 2014; 82:154–163. 10.1002/prot.24495 PMC4495912 24339001

[24] Haas J. , Barbato A., Behringer D., Studer G., Roth S., Bertoni M., Mostaguir K., Gumienny R., Schwede T. Continuous Automated Model EvaluatiOn (CAMEO) complementing the critical assessment of structure prediction in CASP12. Proteins: Struct. Funct. Bioinformatics. 2018; 86:387–398. 10.1002/prot.25431 PMC5820194 29178137

[25] Jiménez J. , Doerr S., Martínez-Rosell G., Rose A.S., De Fabritiis G. DeepSite: protein-binding site predictor using 3D-convolutional neural networks. Bioinformatics. 2017; 33:3036–3042. 28575181 10.1093/bioinformatics/btx350

[26] Le Guilloux V. , Schmidtke P., Tuffery P. Fpocket: an open source platform for ligand pocket detection. BMC Bioinformatics. 2009; 10:168. 19486540 10.1186/1471-2105-10-168 PMC2700099

[27] Zhang Z. , Li Y., Lin B., Schroeder M., Huang B. Identification of cavities on protein surface using multiple computational approaches for drug binding site prediction. Bioinformatics. 2011; 27:2083–2088. 21636590 10.1093/bioinformatics/btr331

[28] Krivák R. , Hoksza D. P2Rank: machine learning based tool for rapid and accurate prediction of ligand binding sites from protein structure. J. Cheminformatics. 2018; 10:39. 10.1186/s13321-018-0285-8 PMC6091426 30109435

[29] Jendele L. , Krivak R., Skoda P., Novotny M., Hoksza D. PrankWeb: a web server for ligand binding site prediction and visualization. Nucleic Acids Res. 2019; 47:W345–W349. 31114880 10.1093/nar/gkz424 PMC6602436

[30] Hernandez M. , Ghersi D., Sanchez R. SITEHOUND-web: a server for ligand binding site identification in protein structures. Nucleic Acids Res. 2009; 37:W413–W416. 19398430 10.1093/nar/gkp281 PMC2703923

