# Supplementary material for: GRaSP-web: a machine learning strategy to predict binding sites based on residue neighborhood graphs
Source: Nucleic Acids Res. 2022 May 7;50(W1):W392–7. doi: 10.1093/nar/gkac323 (PMC9252730; doi:10.1093/nar/gkac323)
Supplement: gkac323_Supplemental_File [file gkac323_supplemental_file.pdf]

GRaSP-web: a machine learning strategy to predict binding sites based on residue neighborhood graphs

Charles A. Santana [1, 2]*, Sandro C. Izidoro [3], Raquel C. de Melo-Minardi [1, 2], Jonathan D.Tyzack [4], António J. M. Ribeiro [4], Douglas E. V. Pires [5], Janet M. Thornton [4] and Sabrina de A. Silveira [6]

[1] Department of Biochemistry and Immunology, Universidade Federal de Minas Gerais, Belo Horizonte, 31270-901,Brazil,
[2] Department of Computer Science, Universidade Federal de Minas Gerais, Belo Horizonte, 31270-901,Brazil,
[3] Institute of Technological Sciences (ICT), Advanced Campus at Itabira, Universidade Federal de Itajubá, Itabira, 35903-087, Brazil,
[4] European Molecular Biology Laboratory, European Bioinformatics Institute, WellcomeTrust Genome Campus, Hinxton, Cambridge CB10 1SD, UK,
[5] School of Computing and Information Systems, University of Melbourne, Parkville, 3052, Australia,
[6] Department of Computer Science, Universidade Federal de Viçosa, Viçosa, 36570-900, Brazil.

*To whom correspondence should be addressed. Email: sabrinas@ufv.br

Table S1

GRaSP results for different datasets. Astex [1] is a dataset of drug-like complexes, including diverse ligands, used to show how our method performs on a diverse and relevant set of drug-like targets. B44/U44 [2] is a dataset of 44 proteins on bound and unbound states used to compare how our method performs on both states. HOLO4K [2] is a benchmark of 4,543 protein-ligand complexes composed of multiple chain structures from PDB used to show that our method can detect binding site residues at protein interfaces.

	ASTEX	B44	U44	HOLO4K
MCC	0.66	0.67	0.67	0.61
Precision	0.74	0.64	0.61	0.68
Recall	0.65	0.77	0.80	0.58

Table S2

Comparative results for GRaSP and methods combined by COACH [3, 4] to create a consensus. COACH combines results of its own algorithms, TM-SITE and S-SITE, with other three third-party ligand-binding site prediction methods, COFACTOR [5], FINDSITE [6] and ConCavity [7], using a supervised learning strategy.

	TM-SITE	S-SITE	COFACTOR	FINDSITE	CONCAVITY	COACH	GRASP
MCC	0.51	0.45	0.46	0.44	0.33	0.60	**0.61**
Precision	0.59	0.45	0.61	0.45	0.26	0.59	**0.69**
Recall	0.51	0.58	0.41	0.51	0.62	**0.70**	0.61

Table S3

Comparison between GRaSP and COACH using B44/U44 dataset. B44/U44 is composed of 44 protein structures in a bound and unbound state and is used to show that our method is able to consistently predict protein-ligand binding site residues for proteins in both states.

Dataset	Method	MCC	Precision	Recall
B44	GRaSP	0.67	0.64	0.77
	COACH	0.64	0.59	0.77
U44	GRaSP	0.67	0.61	0.80
	COACH	0.67	0.75	0.75

References

[1] M. J. Hartshorn *et al.*, ‘Diverse, High-Quality Test Set for the Validation of Protein−Ligand Docking Performance’, *J. Med. Chem.*, vol. 50, no. 4, pp. 726–741, Feb. 2007, doi: 10.1021/jm061277y.

[2] R. Krivák and D. Hoksza, ‘P2Rank: machine learning based tool for rapid and accurate prediction of ligand binding sites from protein structure’, *J. Cheminformatics*, vol. 10, no. 1, p. 39, Dec. 2018, doi: 10.1186/s13321-018-0285-8.

[3] J. Yang, A. Roy, and Y. Zhang, ‘Protein–ligand binding site recognition using complementary binding-specific substructure comparison and sequence profile alignment’, *Bioinformatics*, vol. 29, no. 20, pp. 2588–2595, Oct. 2013, doi: 10.1093/bioinformatics/btt447.

[4] Q. Wu, Z. Peng, Y. Zhang, and J. Yang, ‘COACH-D: improved protein–ligand binding sites prediction with refined ligand-binding poses through molecular docking’, *Nucleic Acids Res.*, vol. 46, no. W1, pp. W438–W442, Jul. 2018, doi: 10.1093/nar/gky439.

[5] A. Roy, J. Yang, and Y. Zhang, ‘COFACTOR: an accurate comparative algorithm for structure-based protein function annotation’, *Nucleic Acids Res.*, vol. 40, no. W1, pp. W471–W477, Jul. 2012, doi: 10.1093/nar/gks372.

[6] M. Brylinski and J. Skolnick, ‘A threading-based method (FINDSITE) for ligand-binding site prediction and functional annotation’, *Proc. Natl. Acad. Sci.*, vol. 105, no. 1, pp.

129–134, Jan. 2008, doi: 10.1073/pnas.0707684105.
[7] J. A. Capra, R. A. Laskowski, J. M. Thornton, M. Singh, and T. A. Funkhouser, ‘Predicting Protein Ligand Binding Sites by Combining Evolutionary Sequence Conservation and 3D Structure’, *PLoS Comput. Biol.*, vol. 5, no. 12, p. e1000585, Dec. 2009, doi: 10.1371/journal.pcbi.1000585.
